# Editorial: Brief Interventions for Risky Drinkers

**DOI:** 10.3389/fpsyt.2016.00042

**Published:** 2016-03-17

**Authors:** Antoni Gual, Hugo López-Pelayo, Jillian Reynolds, Peter Anderson

**Affiliations:** ^1^Grup de Recerca en Addiccions Clínic (GRAC-GRE), Hospital Clínic de Barcelona, IDIBAPS, Red de Trastornos adictivos (RETICS), Barcelona, Spain; ^2^Grup de Recerca en Addiccions Clínic (GRAC-GRE), Hospital Clínic de Barcelona, Fundació Clínic per la Recerca Biomèdica, Red de Trastornos adictivos (RETICS), Barcelona, Spain; ^3^Institute of Health and Society, Newcastle University, Newcastle, UK; ^4^Faculty of Health, Medicine and Life Sciences, Maastricht University, Maastricht, Netherlands; ^5^Centre for Addiction and Mental Health, Toronto, ON, Canada

**Keywords:** alcohol drinking, hazardous drinking, at-risk drinking, brief intervention, brief advice

**The Editorial on the Research Topic**

**Brief Interventions for Risky Drinkers**

Alcohol consumption is a wholly or contributory cause for more than 200 diseases, injuries, and other health conditions with three-digit ICD-10 codes (1). Globally, alcohol is the fifth most important risk factor for ill-health and premature death (2). Risky alcohol use can be defined as a quantity or pattern of alcohol use that places individuals at risk for adverse health and social outcomes (3). Harmful use, in turn, can be defined as alcohol use that results in physical, psychological, or social harm (3). Using a threshold of an average of 60 g of alcohol/day for a man and 40 g/day for a woman (4), about one in four Europeans aged 15–64 years use alcohol in a risky fashion (5). And, using a threshold of an average of 100 g of alcohol/day for a man and 60 g/day for a woman, about one in eight of Europeans aged 15–64 years use alcohol in a harmful fashion (5). Harmful use causes comorbid illnesses such as liver disease, depression, and raised blood pressure (6). Risky and harmful alcohol use and their comorbid illnesses are frequently detected in primary health care, emergency departments, and other non-specialized clinical settings. Brief advice emerged in the 1980s (7–9) and progressed during the three following decades as a strategy to reduce risky and harmful alcohol use in non-specialized clinical settings (10). This article provides an update of the state-of-the art of brief advice.

## Efficacy and Effectiveness of Brief Advice

Twenty-four systematic reviews have demonstrated the efficacy and effectiveness of brief advice delivered in primary health care settings to reduce risky and harmful alcohol use [O’Donnell et al.; (11, 12)]. The negative results found in some studies can be explained by several misconceptions about null findings and should not diminish the strength of the evidence base for the efficacy and effectiveness of brief advice (Heather). Examples of misconceptions include difficulties in distinguishing between “evidence of absence and absence of evidence” and the interference of reduction in consumption in control groups from baseline to follow-up mediated by regression to the mean, a research participation effect, or assessment reactivity.

## Why Does Brief Advice Work?

The underlying mechanisms of the effectiveness of brief advice are only partially known (Gaume et al.). Personalized feedback seems an effective ingredient. Other components (including advice to reduce/stop drinking, presenting alternative change options, moderation strategies, changes in norms perception, discrepancy between current behavior and goals/values, and change plan exercises) appear to be promising. Change talk seems to acts as a mediator of brief advice, whereas readiness to change seems an inconsistent mediator of the effectiveness of brief advice. More research on other potential active ingredients is needed, such as the perceived risk/benefit of alcohol intake, alcohol treatment seeking, self-efficacy, or enhanced awareness.

## For Whom Can Brief Advice Help?

Brief advice seems to work in primary health care and, in emergency departments, for men without other drug use (Wojnar and Jakubczyk). Brief advice does not seem to work for men seen in emergency departments as a consequence of violence-related events, or for women as a whole seen in emergency departments. In general, the effectiveness of brief advice in primary health care for women remains limited (11). Research on the effectiveness of brief advice in social service settings and at the workplace is understudied, and no conclusions of its impact can be made (Schulte et al.). Data on the efficacy of brief advice for illegal drug users are lacking for a number of reasons: concomitant unhealthy alcohol use, comorbid mental health conditions, variety of drugs used, and a wide range in severity (Saitz). In conclusion, there is insufficient evidence to support the implementation of brief advice in settings other than primary health care or for drugs. Further research is needed in these areas.

## Implementation Barriers

Although the cost-effectiveness of brief advice is well-established (Angus et al.), it has not proved a sufficient trigger for the widespread implementation of brief advice in clinical practice, even though key stakeholders in several European health systems (for example, Catalonia, England, Finland, Italy, Scotland, and Sweden) have pushed for it (Colom et al.). Several barriers for implementing brief advice have been identified, including a risk of upsetting patients and a lack of time, training, and incentives (13). This is why a fair share of the current research on brief advice focusses on implementation science, seeking strategies to overcome these barriers.

## Future Lines for Brief Advice

Facilitated access to e-health and m-health modules could potentially boost the implementation and coverage of brief advice, and a number of clinical trials are underway [Wallace and Bendtsen; (14, 15)]. Ambitious projects have already been carried out, such as the FP7 EU funded project ODHIN (www.odhinproject.eu), which compared three strategies for promoting screening and brief advice activity in primary care (training and support, financial reimbursement, and referral to internet-based brief interventions), delivered separately or in combination. The ODHIN project showed the relevance of training and support and of financial incentives to increase the delivery rates of screening and brief advice but failed to find a significant impact of the option of referral to internet-based brief interventions^1^.

Despite the evidence of the effectiveness of brief advice, its uptake in Europe is very low (16). Several authors have recently proposed a new approach to improve dissemination of brief advice for heavy drinking in primary health care (17, 18). Rehm et al. propose a shift from the “prevention approach” to a more medical “treatment approach,” where alcohol problems should be managed with the same strategies and up to the same standards applied for other chronic conditions, such as high blood pressure and diabetes (19). According to this model, special attention should be paid to comorbid conditions such as hypertension, insomnia, liver problems, depression, and anxiety disorders, all of them very prevalent in primary health care.

In conclusion, despite strong evidence on the efficacy, effectiveness, and cost-effectiveness of brief advice in primary health care, its implementation in Europe is still very low. Therefore, new approaches making the best use of new technologies and aiming for a medical management of risky and harmful and alcohol use in primary health care, with the same standards used for common chronic medical conditions, should be tested.

## Author Contributions

All authors have contributed in the writing and intellectual content of the article. All authors have read and approved the manuscript for submission to the journal.

## Conflict of Interest Statement

AG has received grants from Lundbeck, DyA Pharma y TEVA and honoraria from Lundbeck, DyA Pharma and Abbivie that have no relation with the study. HL-P has received honoraria from Lundbeck and Janssen and travel grants from Lundbeck, Lilly, Pfizer, Rovi, and Esteve that has no relation with this work. JR and PA have no conflict of interest to declare.

## Footnotes

^1^Anderson P, Bendtsen P, Spak F, Reynolds J, Drummod C, Segura L, et al. Improving the delivery of brief interventions for heavy drinking in primary health care: outcome results of the ODHIN five country cluster randomized factorial trial. (under review in Addiction).
